# The Presence, Trends, and Causes of Security Vulnerabilities in Operating Systems of IoT’s Low-End Devices

**DOI:** 10.3390/s21072329

**Published:** 2021-03-26

**Authors:** Abdullah Al-Boghdady, Khaled Wassif, Mohammad El-Ramly

**Affiliations:** Department of Computer Sciences, Faculty of Computers and Artificial Intelligence, Cairo University, 5 Ahmed Zewail Street, Dokki, Giza 12613, Egypt; kwassif@fci-cu.edu.eg (K.W.); m.elramly@fci-cu.edu.eg (M.E.-R.)

**Keywords:** internet of things security, internet of things operating systems, C/C++ static analysis, common weakness enumeration, security vulnerability

## Abstract

Internet of Things Operating Systems (IoT OSs) run, manage and control IoT devices. Therefore, it is important to secure the source code for IoT OSs, especially if they are deployed on devices used for human care and safety. In this paper, we report the results of our investigations of the security status and the presence of security vulnerabilities in the source code of the most popular open source IoT OSs. Through this research, three Static Analysis Tools (Cppcheck, Flawfinder and RATS) were used to examine the code of sixteen different releases of four different C/C++ IoT OSs, with 48 examinations, regarding the presence of vulnerabilities from the Common Weakness Enumeration (CWE). The examination reveals that IoT OS code still suffers from errors that lead to security vulnerabilities and increase the opportunity of security breaches. The total number of errors in IoT OSs is increasing from version to the next, while error density, i.e., errors per 1K of physical Source Lines of Code (SLOC) is decreasing chronologically for all IoT Oss, with few exceptions. The most prevalent vulnerabilities in IoT OS source code were CWE-561, CWE-398 and CWE-563 according to Cppcheck, (CWE-119!/CWE-120), CWE-120 and CWE-126 according to Flawfinder, and CWE-119, CWE-120 and CWE-134 according to RATS. Additionally, the CodeScene tool was used to investigate the development of the evolutionary properties of IoT OSs and the relationship between them and the presence of IoT OS vulnerabilities. CodeScene reveals strong positive correlation between the total number of security errors within IoT OSs and SLOC, as well as strong negative correlation between the total number of security errors and Code Health. CodeScene also indicates strong positive correlation between security error density (errors per 1K SLOC) and the presence of hotspots (frequency of code changes and code complexity), as well as strong negative correlation between security error density and the Qualitative Team Experience, which is a measure of the experience of the IoT OS developers.

## 1. Introduction

The Internet of Things (IoT) is a dynamic global network of sensors, actuators, controllers and smart devices that act together to capture, filter and exchange data about their environment, taking advantage of Internet connection and integration capabilities. IoT is a new technology that is growing rapidly and extensively, with an estimated 50 billion devices at the end of 2020 [1]. The combined IoT market will rise to about 520 billion USD in 2021, more than double the 235 billion USD invested in 2017 [2]. Over the last few years, big IoT vendors such as Microsoft (Azure IoT) [3], Amazon (AWS IoT) [4], Cisco (Jasper) [5], Google (Brillo) [6], Apple (Homekit) [7], IBM (Watson) [8], and Qualcomm (AllJoyn) [9] have rapidly grown in the IoT market. Furthermore, over 300 IoT platforms are available today, with more on the way [10]. IoT is a heterogeneous, complex environment and suffers from lack of interoperability. IoT devices are ubiquitous and are sources of big data in terms of their level of size and transmission over the Internet.

IoT devices are therefore a legitimate target for malicious activities, and are highly vulnerable to different kinds of attacks. In order to clarify this point, IoT devices need to interact and exchange the acquired data with IoT platforms for appropriate reactions. As a result, the exchanged data be breached, misplaced or stolen for improper use. For example, health care wearable sensors exchange the health conditions data of patients with smart health platforms to be analyzed and then order appropriate medical assistance. Therefore, Therefore, IoT needs to maintain interoperability with an appropriate level of security. As another example, IoT could be exploited to conduct large-scale Distributed Denial of Service (DDoS) attacks [11,12]. McAfee Threat Report 2017 showed that the Mirai botnet used insecure IoT devices to carry out the largest DDoS attack ever [13]. Hewlett Packard’s study in 2014 revealed that 70% of IoT devices were vulnerable to attack, and that IoT devices averaged 25 vulnerabilities per product [14].

The operating systems of low-end IoT devices (IoT OSs) play a vital role in running and managing IoT devices with respect to sending data through the Internet, taking into account IoT characteristics such as low power consumption, low memory use, and limited computing and storage resources. Popular IoT OSs like RIOT, Contiki, FreeRTOS, and Amazon FreeRTOS are open source and are developed by programmers with different programming backgrounds and different levels of experience when it comes to applying secure coding practices. The number of vulnerabilities reported publicly to the Common Vulnerabilities and Exposures database (CVE) increased from 4500 in 2010 to 18,000 in 2017 [15]. For these reasons, IoT OSs have serious vulnerability issues that need to be taken care of when developing or upgrading IoT OSs.

Static code analysis [16] is a technique for examining the source code against a collection of coding rules without code execution. It is carried out early in the development cycle before software deployment to address code vulnerabilities. Code vulnerabilities are weaknesses in the source code that could lead to security issues and could be exploited by attackers. In the course of this research, source code examination was conducted on sixteen different releases of IoT OS source code in order to increase the awareness level of the security vulnerabilities of IoT OSs by answering our research questions.

***Research Question No. 1***: Do IoT OSs’ security errors increase or decrease as they evolve over time?

***Research Question No. 2***: Does IoT OSs’ security error density, i.e., errors per 1K SLOC increase or decrease as they evolve over time?

***Research Question No. 3***: What are the most prevalent CWE vulnerabilities among IoT OSs?

***Research Question No. 4:*** What is the relationship between the vulnerabilities present in IoT OSs and their evolutionary properties?

The next section of this paper explains the relevant definitions and background knowledge. Section 3 presents the related work on C/C++ errors and vulnerabilities within the source code of low-end IoT device OSs. Then, Section 4 explains the research methodology and case study. Finally, we present the results, discussion, and the conclusion of our research in Section 5, Section 6 and Section 7 respectively.

## 2. Background

This section provides an explanation of the relevant background knowledge directly connected to this research.

### 2.1. Operating Systems of Low-End IoT Devices

IoT devices can be high-end devices that are operated by traditional operating systems, such as Linux, or low-end devices with limited resources, e.g., very limited memory, computational power, and power supply [17]. The scope of this study is low-end IoT OSs, which play a vital role in operating and running low-end devices, taking into account the resource limitations of these devices. We chose four of the popular open-source IoT OSs for this study, based on the following rules. The targeted IoT OS should be: (1) among the most commonly used low-end IoT OSs in the last four years, (2) well documented, (3) open source, and (4) basically developed in C/C++.

We briefly introduce each of the IoT OSs chosen for our study: RIOT [18], Contiki [19], FreeRTOS [20] and Amazon FreeRTOS [21]. They are well documented, developed with C/C++, and are among the most-used open-source IoT OSs, according to the last four surveys of the IoT Eclipse foundation chosen IoT Oss [22,23,24,25]. According to the 2019 IoT Eclipse foundation survey [22], FreeRTOS, Contiki and RIOT accounted for 19%, 5% and 5% of non-Linux operating system use, respectively. Table 1 gives an overview of our case study of IoT OSs.

***RIOT*** is an open-source real-time operating system for low-end IoT devices built on Microkernel architecture [26]. A grassroots community gathering companies, academics, and hobbyists, distributed all around the world, developed RIOT. It was mainly written from scratch using the C/C++ programming language, with minor use of other languages such as Python, and it seeks to implement all related open standards supporting IoT. RIOT was developed to use minimal resources in terms of power consumption, ROM (~5 kB), and RAM ((~1.5 kB) [27]. RIOT offers a generic API to access sensor and actuator devices, named the Sensor Actuator Uber Layer (SAUL) API. This API enables a vendor-agnostic access to sensors and actuators and allows applications to be written against heterogeneous IoT devices using the same function calls. RIOT runs on a variety of platforms, such as embedded devices and personal computers.

***Contiki*** has a Monolithic architecture [28], and was especially designed to run low-end IoT devices. It makes it possible to build applications that allow effective use of hardware while ensuring adequate low-power wireless communication for a variety of hardware platforms, where it enables microcontroller chips to connect to the Internet. It is mainly implemented in C with minor use of other languages such as Python and Java, Contiki prioritizes light power performance and memory management, with standard setups implemented using as little as 2 KB of RAM and 60 KB of ROM running at 1 MHz [29]. Popular SSL/TLS libraries such as wolfSSL support and perfectly match Contiki operating system, which was developed with portability in mind [30].

***FreeRTOS*** has a Microkernel architecture [28] and was basically written in the C programming language over 15 years in collaboration with the world’s leading chip companies. Its focus was reliability and ease of use. It is distributed free of charge under the Massachusetts Institute of Technology (MIT) open-source license. FreeRTOS consumes less than 4 to 9 KB of ROM and provides a collection of libraries for handling File Allocation Table (FAT) and storage media. Therefore, it is efficient for running low-end IoT devices.

***Amazon FreeRTOS.*** Amazon provides an expansion of FreeRTOS, referred to as Amazon FreeRTOS and is a Microkernel architecture. Amazon FreeRTOS was basically written in the C programming language with minor use of other languages such as Python, Perl and Ruby. FreeRTOS includes libraries for IoT support, and is specifically for Amazon Web Services (AWS). Since version 10.0.0 (2017), Amazon has been in charge of the FreeRTOS’s source code, including any changes to the original kernel.

### 2.2. Common Weakness Enumeration Vulnerabilities

Software vulnerabilities are weaknesses in the source code. Vulnerabilities stem from insecurities in the language used, combined with ignoring secure coding practices by the programmers, the pressure of deadlines, and/or lack of management focus on the topic [31]. Since IoT integrates multiple devices, sensors, and actuators and interacts directly with humans in many of its applications, the presence of vulnerabilities in IoT systems can have severe consequences. Imagine, for example, hacking a pacemaker device or a self-driving car [14]. The situation is further complicated by the fact that most IoT OSs are written in C/C++ due to their very powerful low-level programming support. However, at the same time, they are among the least secure programming languages. Some studies claim that 50% of vulnerabilities in open-source projects discovered between 2009 and 2019 were in C programs [32].

Common Weakness Enumerations vulnerabilities (CWEs) [33] is a community-developed evolving formal list of software vulnerability types, called weaknesses. The CWE list and related classification taxonomy act as vocabulary that can be used in terms of CWEs to define and explain these vulnerabilities. The main objective of CWE is to avoid vulnerabilities in the source code by educating software and hardware programmers, designers, architects about how to remove the most common errors before software and hardware are delivered, targeted at both the development and security communities.

### 2.3. Code Analysis Tools

To analyze the chosen IoT OSs in our study of the presence of vulnerabilities, Static Analysis Tools (SATs) were used to target and identify vulnerabilities in the source code without being executed. Cppcheck version 2.1 [34], Flawfinder version 2.0.11 [35], and Rough Auditing Tool for Security (RATS) [36] were our chosen SATs for examining the IoT OS source code. The three SATs are well documented, free, well known among the research community [37,38,39], and CWE compatible. Besides the three SATS, we needed a tool to examine the evolution of the IoT OS code base and the factors that affect its well-being over time in order to study the factors that may influence the existence of vulnerabilities. For this purpose, we used CodeScene [40] to investigate the evolutionary properties of IoT OSs. We introduce each of these tools as the following.

***Cppcheck*** is a C/C++ static analysis tool. It provides comprehensive code analysis to find errors of source code, focusing on the identification of unknown actions and unsafe code, such as divide by zero, dead pointers, null pointer dereferences to name and integer overflows. Cppcheck is designed to analyze the source code and classify the severity of the errors found. The tool locates errors and potential errors, issuing messages identifying errors, giving warnings with recommendations to prevent errors, suggesting performance recommendations for faster code, etc. Our analysis results focus on vulnerabilities related to CWE, since this is our work benchmark.

***Flawfinder*** is an open-source tool used to look for potential security errors within C/C++ source code and it is officially CWE-Compatible. Flawfinder investigates source code, categorizing the findings from level 0, a very little level of risk, to 5, a high level of risk, ignoring text inside comments and strings. Flawfinder is highly sensitive to error detection, and the author of Flawfinder stated that “Not every hit is necessarily a security vulnerability” at the end of Flawfinder result reports.

***RATS*** is an open-source tool that has the capability of scanning various programming languages such as C, C++, Perl, PHP and Python source code. RATS flags common programming errors related to security, such as buffer overflows and TOCTOU (Time Of Check, Time Of Use) race conditions.

**Figure 1:**
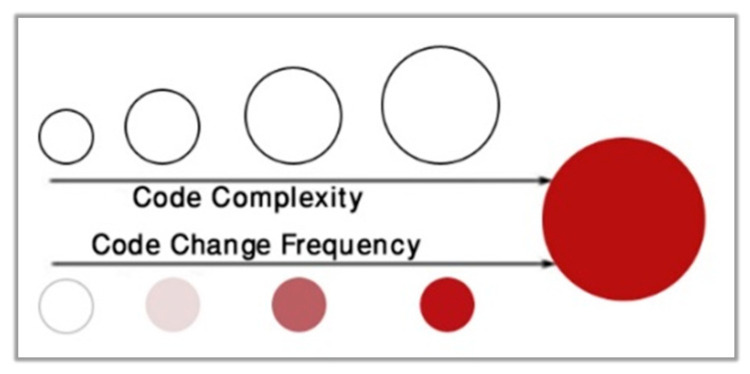
Hotspot calculation of the code.

***CodeScene***: By analyzing the evolution of the code, CodeScene [41] detects trends at the level of the entire system and at the file level. CodeScene can track code Hotspots, which are the complicated pieces of code that developers often have to work with. Hotspots are determined by combining the change frequency of each file as an interest rate proxy and the code lines as a simple measure of code complexity. Consequently, Hotspot analysis finds those files where much of the development time is spent. As shown in Figure 1, the darker the red color is, the more commits (changes) that have been done to the code. The wider the circle is, the wider circle is, the more the code it represents in the file.

CodeScene can also track Code Health, which refers to the ease of code maintenance and evaluation. The Code Health metric is calculated on the basis of a combination of both the properties of the code and the organizational factors, with a total of 25–30 biomarkers of code depending on the programming language. Some of the biomarkers used in calculating Code Health include (1) Brain Methods “single functions/methods that center too much behavior”, (2) Nested complexity “such as if statements inside other if statements and/or loops”, (3) Do not Repeat Yourself (DRY) violations, (4) Bumpy Road “a function that fails to encapsulate its responsibilities”, and (5) Developer Congestion, “code becomes a coordination bottleneck when multiple developers need to work on it in parallel” [40].

**Figure 2:**
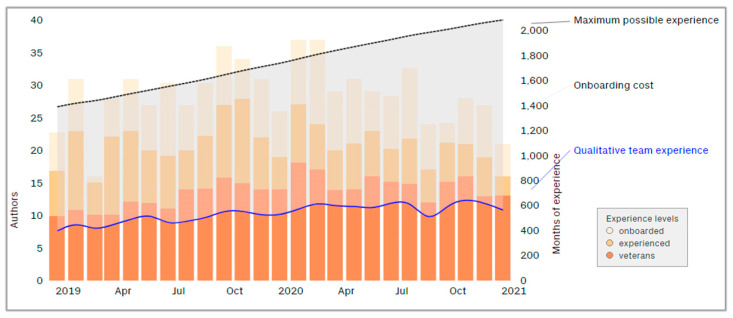
CodeScene representation of RIOT team composition.

Additionally, in terms of experience, CodeScene can calculate the monthly composition of the team. CodeScene classifies the experiences of teams into three categories: on-board (0–3 months), seasoned (6–12 months), and veterans (+12 months). Figure 2 shows the RIOT team composition from 2019 to 2021, where the total accumulated experience in terms of months (black line) and “Qualitative” Team Experience is a weighted value in which the experience of each developer currently in the team is taken into account (blue line).

## 3. Related Work

Table 2 shows a brief overview of the related work, and the following paragraphs explain the work in details. Alnaeli et al. [42] conducted an empirical study using static analysis methods on three C/C++ open-source IoT software packages to identify known vulnerable statements. They created a tool called UnsafeFunsDetector to find unsafe functions that are known to the research community or banned by some compiler producers. The study found that vulnerable functions were very common among the three systems, where memcpy() was the most prevalent unsafe function, followed by strlen(). The number of unsafe functions increased over the five-year period from 2012 to 2016 for the studied systems, reaching 1859 unsafe functions for Contiki OS, 772 unsafe functions for TinyOS, and 220 unsafe functions for OpenWSN.

Alnaeli et al. [43] extended their previous work [42] to empirically examine the vulnerabilities of eighteen open-source IoT software systems, all of which were specifically written in C/C++ for IoT architectures. They found that usage of unsafe functions was still common among the selected systems, and developers were not working to improve the problems that were still present in the selected systems. On the other hand, memcpy() was the most prevalent unsafe function in the majority of the systems, followed by strlen(), free(), and strcmp(). In both studies, Alnaeli et al. focused on known vulnerable statements, ignoring many types of IoT OSs vulnerabilities that could lead to serious security issues.

McBride et al. [44] conducted a study using static program analysis tools and techniques to scan the Contiki OS source code in order to identify errors, bug density, and unsafe functions. The study found an obvious increment of unsafe functions over the past 10 years of releases for Contiki OS. Unsafe functions increased from Contiki Version 2.0, with a total of 334 unsafe functions, to 1311 unsafe functions in Contiki Version 3.x, where memcpy() was the most prevalent unsafe statement, with 743 unsafe functions, followed by strlen(), with 375 functions.

Both Alnaeli et al. [42,43] and McBride et al. [44] presented different results for the total number of unsafe functions, since different static analysis tools were used. Both of the studies used the term unsafe function to describe vulnerable commands and statements in terms of functions written by C/C++ programming language. Nevertheless, Contiki contains some files written using the Python programming language, and these files suffer from security errors; both Alnaeli et al. [42,43] and McBride et al. [44] ignored these errors.

Mullen and Meany [45] conducted a comprehensive assessment of Buffer Overflow (BOF) attacks, one of the most prevalent vulnerabilities in IoT devices running an IoT OS. The assessment was conducted for IoT devices use FreeRTOS version 9.0.0, focusing on two such attacks, namely return-to-libc and code injection. The assessment addressed the mechanics, implementation, and testing of BOF attacks and how to prevent them. It also exposed the limitations of FreeRTOS with respect to BOF prevention methods.

Mahmood and Mahmoud [46] conducted an evaluation on SATs for finding vulnerabilities in Java and C/C++ source code. They explained that none of the studied tools was sufficient to comprehensively uncover all present vulnerabilities. They recommended the adoption of secure coding techniques and the use of several vulnerability detection methods to reduce source code security risks.

Our work adopts this approach by employing multiple SATs. We complement the previous studies by further investigating the presence of vulnerabilities in IoT OSs up to their 2020 versions. However, we differ in taking a broader approach by examining multiple systems over multiple versions, up until the most recent ones, using multiple SATs. Furthermore, we take CWEs as the benchmark for identifying vulnerabilities and we use CodeScene to study IoT OSs’ Code Health and factors that affect the presence of vulnerabilities.

## 4. Methodology

**Figure 3:**
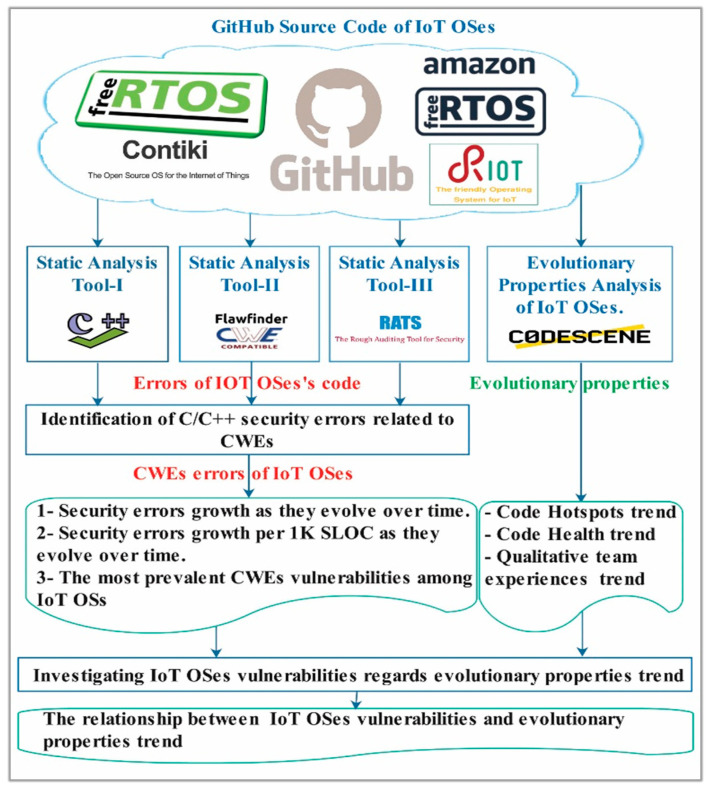
Methodology of finding and investigating IoT OSs security vulnerabilities.

Figure 3 illustrates the methodology of our research investigating the presence of security vulnerabilities in IoT OSs. The study targeted sixteen releases of the four previously mentioned IoT OSs from 2010 to 2020. Source code was obtained from the GitHub repository of each of each IoT OSs. Table 3 shows the targeted IoT OS releases and their corresponding year of release.

Cppcheck version 2.1 [34], Flawfinder version 2.0.11 [35], and RATS version 2.4 [36] were used to examine and identify errors in the IoT OSs’ source code that could lead to security vulnerabilities. The output report of the three SATs describes errors that leads to a security vulnerability with “Error”; thus, our study refers to security vulnerabilities as errors when mentioning the total number of errors and errors per 1K SLOC of IoT OSs. The source code of each IoT OS release was examined standalone by the three SATs, with 48 examinations for all IoT OS releases. The methodology targeted C/C++ files and errors related to the CWE list, identifying the CWE errors of each IoT OS release and creating a report of SAT results. This step aimed to find the growth of total number of errors, errors per 1K SLOC, and the most prevalent CWE vulnerabilities of IoT OSs.

Consequently, CodeScene was used to investigate the relationship between the growth of total number of errors, errors per 1K SLOC, and the development evolutionary properties trend of IoT OSs. For this step, and based on the three SATs results, two IoT OSs were nominated to be examined by CodeScene. The first nominated IoT OS was the one with the lowest errors per 1K SLOC, where the second nominated IoT OS is the one with the highest errors per 1K SLOC. Finally, answers for the four research questions are provided.

## 5. Results

We start by discussing the results of running SATs on the target systems. The three SATs obviously produced different results at the level of CWE error ID, the total number of errors, and the number of errors per 1K SLOC, because each SAT is designed to detect certain errors and applies certain rules for security error detection. The following subsections illustrate the examination results of each IoT OS.

### 5.1. RIOT Examination Results

**Figure 4:**
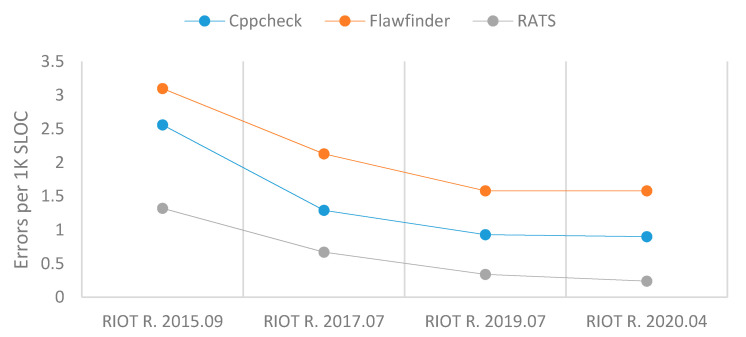
Errors per 1K SLOC of RIOT.

Table 4 illustrates that the total number of errors found by the three SATs increased over time, despite a little decrease from RIOT R. 2017.07 and RIOT R. 2020.04 by RATS examination. Table 4 and Figure 4 show that errors per 1K SLOC decreased chronologically according to the three SATs. While these results suggest that a significant number of errors was still present in RIOT’s latest version at the time of the study, they also show a significant improvement in the error trend relative to 1K SLOC.

### 5.2. Contiki Examination Results

**Figure 5:**
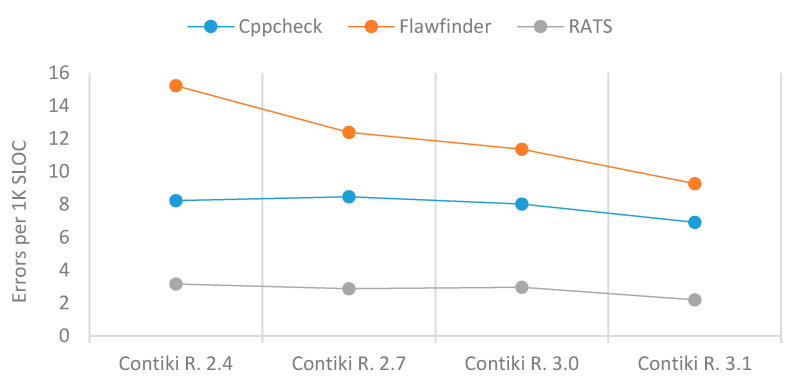
Errors per 1K SLOC of Contiki.

All three SATs showed that the total number of errors in Contiki increased from one version to the next, and a significant number of errors was still present in Contiki’s latest version at the time of the study, as shown in Table 5. Nevertheless, the number of errors per 1K SLOC was chronologically decreased slightly, but this was improved significantly in the latest version as shown in Table 5 and Figure 5.

### 5.3. FreeRTOS Examination Results

**Figure 6:**
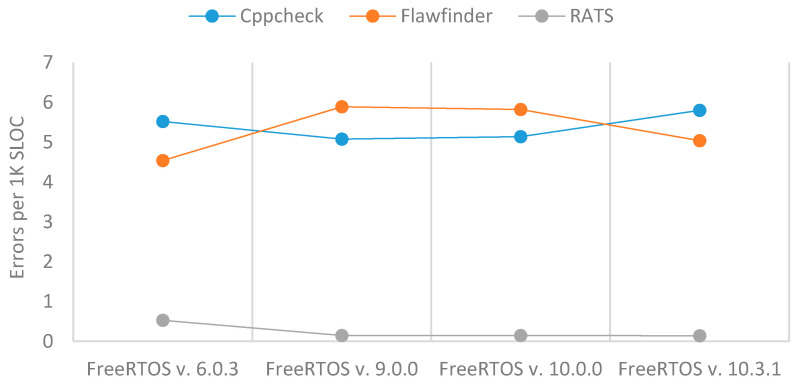
Errors per 1 K SLOC of FreeRTOS.

Table 6 and Figure 6 show that the total number of errors in FreeRTOS increased over time according to the three SATs, except for FreeRTOS version 10.3.1 scanned by Flawfinder. Unlike RIOT, which saw significant improvements in code security over time, the number of errors per 1K SLOC in FreeRTOS stayed more or less the same.

### 5.4. Amazon FreeRTOS Examination Results

**Figure 7:**
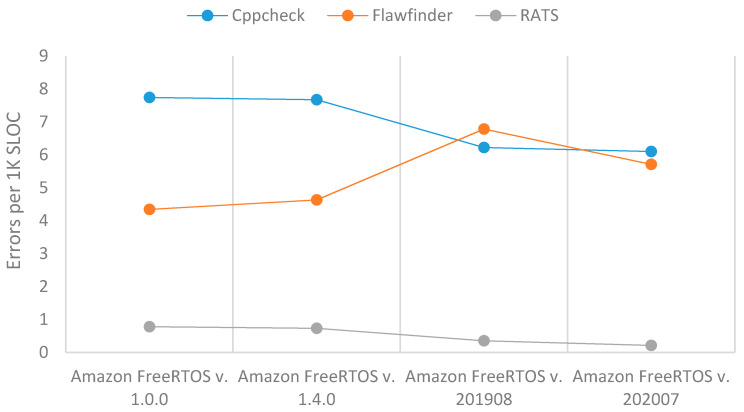
Errors per 1K SLOC of Amazon FreeRTOS.

As can be seen in Table 7 and Figure 7, the three SATs reveal that the total number of errors increased until version Amazon FreeRTOS v. 201908, after which it significantly decreased in version Amazon FreeRTOS v. 202007. The decrease in total number of errors was due to a decrease in SLOC from Amazon FreeRTOS v. 201,908 to Amazon FreeRTOS v. 202007. In addition, this IoT OS had the least number of vulnerabilities per 1K SLOC except by Flawfinder, which showed similar behavior to FreeRTOS.

### 5.5. The Most Common Vulnerabilities in IoT OSs

Table 8, Table 9 and Table 10 show the total number of errors and the error frequency for each IoT OS release with respect to the CWEs that are found by the three SATS. From these tables, we can see that the most prevalent vulnerabilities in the IoT OSs according to Cppcheck 2.1 were CWE-561, CWE-398 and CWE-563, where (CWE-119!/CWE-120), CWE-120 and CWE-126 were the most prevalent vulnerabilities according to Flawfinder 2.0.11, and CWE-119, CWE-120 and CWE-134 were the most prevalent vulnerabilities according to RATS 2.4. The description of the CWEs is set out in Appendix A.

### 5.6. Investigating Evolutionary Properties of the IoT OSs using CodeScene

From Table 4, Table 5, Table 6 and Table 7, it is clear that RIOT has the lowest error rate, and Contiki has the highest, while FreeRTOS and Amazon FreeRTOS are in between. Additionally, the numbers of errors per 1K SLOC for RIOT and Contiki were clearly high. For these reasons, RIOT and Contiki served as our case study for CodeScene. To investigate the causes of these findings, CodeScene was used to examine the effect of the evolutionary properties of IoT OSs, such as Hotspots, Code Health, Qualitative Team Experiences on the total number of security errors and number of security errors per 1K SLOC.

#### 5.6.1. Investigation of the Evolutionary Properties of RIOT

As shown in Table 11, CodeScene addresses the decline of Hotspots across RIOT releases, and there have been high development efforts and bug fixes within the RIOT Hotspots area. CodeScene also shows that the Qualitative Team Experience increased across RIOT releases. These results explain the decline in errors per 1K SLOC according the three SATs’ examinations of RIOT.

In another direction, CodeScene shows that the source code for RIOT releases was healthy. Despite the decline of Code Health across ROIT releases, Code Health value remains higher than 6 out of 10, and thus the total number of errors of RIOT releases was not as high. Nevertheless, it should alarming to project developers that Code Health is declining from a version to the next.

#### 5.6.2. Investigation of the Evolutionary Properties of Contiki

As shown in Table 12, CodeScene shows that Contiki releases contain a higher percentage of code Hotspots than RIOT and high development efforts and bug fixes within Hotspots. Therefore, Contiki releases suffer from code errors and vulnerabilities. CodeScene shows a decline of Hotspots and increase of Qualitative Team Experiences across Contiki releases. This explains the decline in error rate, i.e., errors per 1K SLOC by the three SATs’ examinations of Contiki as in Table 5. Table 12 also shows that Contiki releases were not healthy, the value of Code Health is always less than 5 out of 10, and is declining from a version to the next. Thus, the total number of errors of Contiki releases were high.

**Figure 8:**
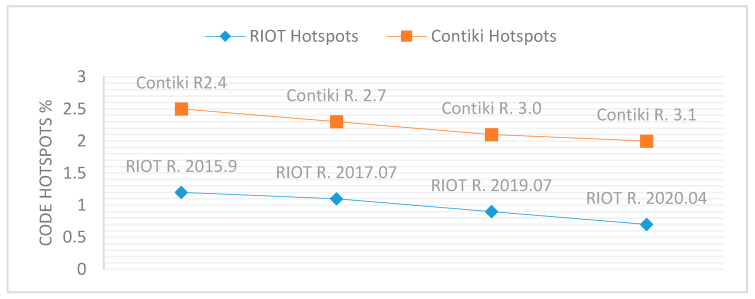
Hotspot evolution of RIOT and Contiki releases.

**Figure 9:**
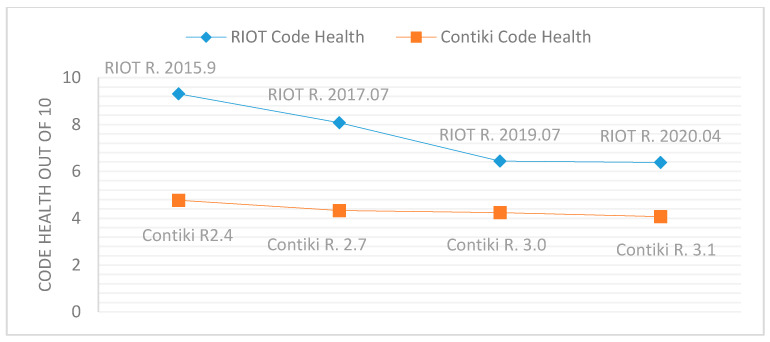
Code Health evolution of RIOT and Contiki releases.

**Figure 10:**
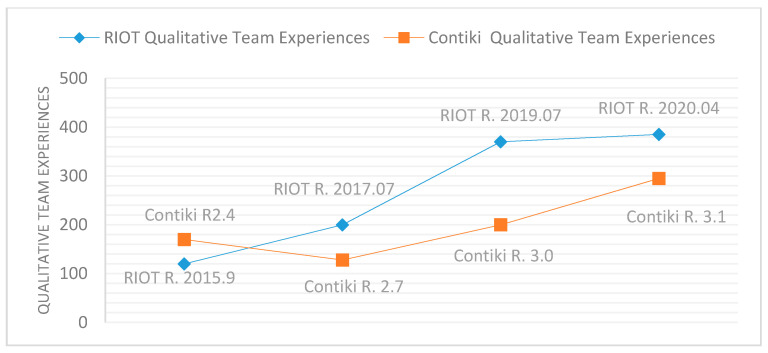
Qualitative Team Experiences evolution of RIOT and Contiki releases.

Figure 8, Figure 9 and Figure 10 provide comparisons between RIOT and Contiki releases based on Hotspots, Code Health, and Qualitative Team Experience. These comparisons show that RIOT releases had fewer Hotspots, higher Code Health, and higher Qualitative Team Experience than Contiki releases. The high value of Contiki’s Hotspots indicates high code change frequency and high code complexity, and the low value of Contiki’s Code Health means that the development of Contiki violated healthy code metrics such as the Brain Method, Nested Complexity, DRY, Bumpy Road, and Developer Congestion. In addition, Contiki does not set up security policies for its development repository [47], where RIOT activates security policies [48]. Therefore, RIOT releases showed the best results according to the three SATs.

For more clarifications, we expressed the relationship between the evolutionary properties and the presence of IoT OS vulnerabilities by calculating the linear correlation coefficient between security errors and code evolutionary properties, as shown in Table 13. The values of X were obtained from Table 4 and Table 5, where values of Y were obtained from Table 3, Table 11 and Table 12. If the correlation coefficient is closer to 1, it implies a strong positive relationship, where a strong negative relationship between the two variables is indicated if it is close to −1. A value of zero means that a relationship does not exist.

From Table 13, there is a strong positive correlation between the total number of security error within IoT OSs and SLOC, as well as a strong negative correlation between the total number of security errors and Code Health. Table 13 also indicates a strong positive correlation between the number of security errors per 1 K SLOC and the presence of Hotspots (frequency of code change and complexity of code), as well as a strong negative correlation between the number of security errors per 1 K SLOC and the Qualitative Team Experience.

## 6. Discussion

After investigating the results of the three SAT analyses, the results were different in terms of the level of CWE error IDs, total number of errors, and number of errors per 1K SLOC. In analyzing these SATs, we found that each SAT applied certain rules for error detection and specialized in detecting certain CWEs. Nevertheless, among the three tools, RATS had the ability to detect errors within IoT OSs’ files written using the Python, Perl, and Ruby scripting languages. However, these errors were neglected, because the study focused on C/C++ IoT OSs files in order to have a fair comparison between the three IoT OSs. The neglected errors included 513 errors in versions of Amazon FreeRTOS, 46 errors in RIOT releases, 46 errors in Contiki releases, and zero errors in versions of FreeRTOS. In the following, we provide the answers to our research questions.

***Research Question No. 1***: Do IoT OSs’ security errors increase or decrease as they evolve over time? ***The answer is***: Except for the latest version of Amazon FreeRTOS, IoT OSs show steady growth in the total number of security errors. This is primarily due to the growth in size. These results suggest that there are ample chances for attackers to exploit such systems, and that the problem is increasing over time.

***Research Question No. 2***: Does IoT OSs’ security error density, i.e., errors per 1K SLOC increase or decrease as they evolve over time? ***The answer is***: Generally, and with few exceptions, there was a gradual decline in the number of errors per 1K SLOC, suggesting an improvement in code security and better use of secure coding practices. However, if we consider Flawfinder, as it is CWE compatible, and we take the latest version examined from each IoT OS in this study, we find that the security errors densities in RIOT, Contiki, FreeRTOS, and Amazon FreeRTOS were 1.58, 9.26, 5.04, and 5.71 vulnerabilities per 1K SLOC, respectively. First, we see big variations, with the highest (Contiki) being roughly 6 times the lowest (RIOT). This suggests that the use of secure code practices among IoT OS developers varies significantly. Second, if we average these figures, we get about 5.4 errors per 1K SLOC (a similar result is obtained if we take the median value), which is still a significant rate. Developers of IoT OSs need to be much more aware of this topic and employ secure code practices and development life cycle. RIOT’s superior results are easily explained by the adoption of security policies in the project, according to the project’s owners [48].

***Research Question No. 3***: What are the most prevalent CWE vulnerabilities among IoT OSs? ***The answer is***: CWE-561, CWE-398 and CWE-563 were Cppcheck’s most prevalent vulnerabilities. For Flawfiner, these were (CWE-119!/CWE-120), CWE-120 and CWE-126. For RATS, they were CWE-119, CWE-120 and CWE-134.

CWE-561 means that the software contains dead code that can never be executed. During code evolution and maintenance, dead code can lead to confusion and result in vulnerabilities. CWE-398 is one of the phyla classifications in the Seven Pernicious Kingdoms vulnerability classification [49]. It does not introduce a weakness or vulnerability directly, but indicates that the software has not been carefully developed or maintained, and this increases the possibility of buried vulnerabilities within the code. CWE-398 refers to an unused variable, and a bug can be pointed out.

CWE-119 refers to memory corruption, where the software fails to constrain operations within the memory buffer boundaries. Consequently, the attackers may execute arbitrary code, sensitive data may be read, or the software may crash. CWE-120 is a classic buffer overflow, where the program copies the buffer without checking its length at all, neglecting the most fundamental security protections. CWE-126 is a buffer over-read, which means that the program reads from a buffer using buffer access mechanisms, which reference memory locations after the targeted buffer. This leads to sensitive information being exposed, or possibly a program crash. CWE-134 is an uncontrolled format string; this could lead to buffer overflows or problems with data representation.

***Research Question No. 4***: What is the relationship between the presence of vulnerabilities and the evolutionary properties of IoT OSs? ***The answer is***: CodeScene shows that the low Code Health of IoT OS leads to a high number of total security errors. In addition, the three SATs show that the total number of security errors of IoT OSs generally increase or decrease due to an increase or decrease in SLOC. CodeScene also shows that the decline of errors per 1K SLOC depends on the decline of code Hotspots and the increase in Qualitative Team Experience. The take-away from this analysis is that vulnerabilities in IoT OSs increase with the increase of Hotspots, which refers to the increase of code change frequency and code complexity. Moreover, vulnerabilities decrease with the increase of Qualitative team experience and Code Health (ease code maintenance and evolution). Additionally, the violation of healthy code metrics such as the Brain Method, Nested Complexity, DRY, Bumpy Road, and Developer Congestion leads to high numbers of vulnerabilities. Furthermore, activating security policies of IoT OSs development repository could help decrease the vulnerabilities.

The limitations of our study are as the following. (1) The IoT OSs of our study were written using various programing languages such as C, C++, Python, Perl, Ruby and Java, where the study SATs are able to perform static analysis only on C/C++ files with the exception of RATS. (2) The study depends on non-commercial SATs and code analysis tools which have limitations. The produced results are limited by the limitations of these tools. While the SATs used are able to find a wide range of CWEs, they are not perfect and may not catch all present vulnerabilities. For example, Cppcheck can detect 83.5% of vulnerabilities, and has 7.2% of false alarms [37]. The results of Flawfinder are close to the RATS results, where Flawfinder works by matching simple text patterns, which results in many false positives [37,46]. CodeScene only illustrates the complexity metric of code Hotspot by simple complexity metric (LOC), ignoring other important complexity metrics such as Cyclomatic complexity. Finally, (3) We considered only three factors when investigating the causes of vulnerabilities in IoT OSs, which are Hotspots (code complexity and change frequency), Code Health (maintainability) and Qualitative Team Experience, which are the main metrics supported by CodeScene. While these are very reasonable factors to study, other factors may influence the presence and trends of vulnerabilities, e.g., process followed, security policies set, team size and hierarchy, etc.

## 7. Conclusions and Future Work

IoT OSs still suffer from errors that could lead to security vulnerabilities, and the total number of errors increases chronologically across IoT OSs’ releases. The good news is that errors per 1K SLOC decreased chronologically for all IoT OS releases, with few exceptions. The exceptions were that error rate increased among FreeRTOS’s versions when examined by Cppcheck and Flawfinder. It also increased among Amazon FreeRTOS’s versions upon examination by Flawfinder.

The three SATs produced different results at the level of CWE IDs, total number of errors, and number of errors per 1K SLOC, because each SAT applies certain rules for error detection and specializes in detecting certain things.

CWE-561, CWE-398 and CWE-563 were the Cppcheck’s most prevalent vulnerabilities in the IoT OS source code, where (CWE-119!/CWE-120), CWE-120 and CWE-126 were Flawfinder’s, and CWE-119, CWE-120 and CWE-134 were RATS’es.

The three SATs show that the total security errors of IoT OSs are generally dependent on the growth of SLOC. Investigating the evolutionary properties of the IoT OSs by CodeScene shows that low Code Health of IoT OSs leads to a high number of total security errors, and the decline of errors per 1 K SLOC depends on the decline of code Hotspots and the increase in Qualitative Team Experience.

Finally, we can conclude that one standalone SAT could not cover all vulnerabilities, and it is recommended to use various SATs to cover a wide range of vulnerability detections. In addition, SATs produce different results at the level of CWE ID and total discovered errors. Hotspots, Code Health, and Qualitative Team Experience are important evolution factors that developers should take care of during the development phase. Hence, IoT OS sponsors should make clear use of security policies during the Software Development Life Cycle (SDLC) by using various SATs, by preventing or raising awareness of the use of known unsafe functions such as memcpy(), strlen(), free(), and strcmp(), by training team developers, by encouraging code documentation, and by encouraging security testing prior to release.

Our immediate future work will extend the use of SATs to identify security errors within IoT OS files written not only by C/C++ but also by other languages such as Python, Perl, and Ruby scripting. Furthermore, our case study will be extended to include IoT OSs such as TinyOS, OpenWSN and Femto OS.

As we contribute new research results to the study of security of IoT OSs for low-end devices, there is still a need for multiple further studies. First, similar studies are needed for other IoT OSs not included in this study. Second, similar studies are needed for popular commercial and open-source IoT applications and systems, other than OSs. Third, while we touched on the underlying factors that can contribute to the presence of vulnerabilities in IoT OSs through our analysis using CodeScence, further and deeper investigations of the root causes are still needed. Practices and project characteristics that lead to vulnerabilities are needed. Fourth, we focused on IoT OSs written in C/C++, which are the dominant languages in this domain, but other languages are also used and can be sources of vulnerabilities. Fifth, and most important, while advanced and sophisticated SATs help expose vulnerabilities, similarly advanced tools need to be developed for vulnerability remediation and automatic program repair.

## Figures and Tables

**Table 1:** Overview of the case study of low-end IoT OSs.

IoT OS	Open Source	Well-Documented over GitHub Repository	Basic Programing Languages	Architecture
RIOT	Yes	Yes	C/C++	Microkernel
Contiki	Yes	Yes	C	Monolithic
FreeRTOS	Yes	Yes	C	Microkernel
Amazon FreeRTOS	Yes	Yes	C	Microkernel

**Table 2:** Brief overview of related work.

Author	IoT OS/Dataset	Years	SAT/Methodology	Scope
1st study of Alnaeli et al.	OpenWSN, Contiki, TinyOS	2012–2016	UnsafeFunsDetector	Find the known vulnerable statements in IoT OSs
2nd study of Alnaeli et al.	ApacheMyNewtOS, AtomThreads, Contiki, DistortOS, Embox, FemtoOS, FreeOSEK, Lepton, nOS, OpenTag, OpenWSN, PicoOS, POK, TinyOS, Tneo, Trampoline, uOS-Embedded, Zephyr	2012–2017	UnsafeFunsDetector	Find the known vulnerable statements in IoT OSs
McBride et al.	Contiki	2007–2018	CodeSonar, Understand, Cppcheck, Clang, Flawfinder, RATS	Find errors and bug density in IoT OSs
Mullen and Meany	FreeRTOS version 9.0.0	2016	Implementation of known BoF attack to assess the BoF prevention mechanism of IoT OS	Comprehensive assessment of BoF attack in IoT OSs
Mahmood and Mahmoud	Software Assurance Metrics and Tool Evaluation (SAMATE) Project website	2018	Yasca, RATS and Flawfinder for C/C++	Evaluation of SATs on finding vulnerabilities

**Table 3:** Case study of IoT OSs releases.

OS	Release or Version	Year	SLOC
RIOT	RIOT R. 2015.09	2015	297,364
	RIOT R. 2017.07	2017	797,714
	RIOT R. 2019.07	2019	1,530,460
	RIOT R. 2020.04	2020	1,788,385
Contiki	Contiki R. 2.4	2010	148,552
	Contiki R. 2.7	2013	240,774
	Contiki R. 3.0	2015	265,083
	Contiki R. 3.1	2017	361,156
FreeRTOS	FreeRTOS v. 6.0.3	2010	650,380
	FreeRTOS v. 9.0.0	2015	2,486,601
	FreeRTOS v. 10.0.0	2017	2,524,568
	FreeRTOS v. 10.3.1	2020	2,808,922
Amazon FreeRTOS	Amazon FreeRTOS v. 1.0.0	2017	401,997
	Amazon FreeRTOS v. 1.4.0	2018	931,240
	Amazon FreeRTOS v. 201908	2019	2,478,140
	Amazon FreeRTOS v. 202007	2020	2,053,453

**Table 4:** Total errors and errors per 1K SLOC of RIOT.

SAT	Release or Version	Total Errors	Errors per 1 K SLOC
Cppcheck	RIOT R. 2015.09	762	2.56
	RIOT R. 2017.07	1026	1.29
	RIOT R. 2019.07	1421	0.93
	RIOT R. 2020.04	1616	0.90
Flawfinder	RIOT R. 2015.09	923	3.1
	RIOT R. 2017.07	1702	2.13
	RIOT R. 2019.07	2415	1.58
	RIOT R. 2020.04	2815	1.58
RATS	RIOT R. 2015.09	394	1.32
	RIOT R. 2017.07	534	0.67
	RIOT R. 2019.07	515	0.34
	RIOT R. 2020.04	436	0.24

**Table 5:** Total errors and errors per 1 K SLOC of Contiki.

SAT	Release or Version	Total Errors	Errors per 1 K SLOC
Cppcheck	Contiki R. 2.4 (2010)	1222	8.23
	Contiki R. 2.7 (2013)	2037	8.46
	Contiki R. 3.0 (2015)	2127	8.02
	Contiki R. 3.1 (2017)	2494	6.91
Flawfinder	Contiki R. 2.4 (2010)	2261	15.22
	Contiki R. 2.7 (2013)	2980	12.38
	Contiki R. 3.0 (2015)	3010	11.35
	Contiki R. 3.1 (2017)	3345	9.26
RATS	Contiki R. 2.4 (2010)	470	3.16
	Contiki R. 2.7 (2013)	691	2.87
	Contiki R. 3.0 (2015)	713	2.96
	Contiki R. 3.1 (2017)	791	2.19

**Table 6:** Total errors and errors per 1K SLOC of FreeRTOS.

SAT	Release or Version	Total Errors	Errors per 1 K SLOC
Cppcheck	FreeRTOS v. 6.0.3 (2010)	3591	5.52
	FreeRTOS v. 9.0.0 (2015)	12,637	5.08
	FreeRTOS v. 10.0.0 (2017)	12,976	5.14
	FreeRTOS v. 10.3.1 (2020)	16,293	5.8
Flawfinder	FreeRTOS v. 6.0.3 (2010)	2951	4.54
	FreeRTOS v. 9.0.0 (2015)	14,655	5.89
	FreeRTOS v. 10.0.0 (2017)	14,704	5.82
	FreeRTOS v. 10.3.1 (2020)	14,166	5.04
RATS	FreeRTOS v. 6.0.3 (2010)	342	0.53
	FreeRTOS v. 9.0.0 (2015)	373	0.15
	FreeRTOS v. 10.0.0 (2017)	374	0.15
	FreeRTOS v. 10.3.1 (2020)	397	0.14

**Table 7:** Total errors and errors per 1K SLOC of Amazon FreeRTOS.

SAT	Release or Version	Total Errors	Errors per 1 K SLOC
Cppcheck	Amazon FreeRTOS v. 1.0.0 (2017)	3113	7.74
	Amazon FreeRTOS v. 1.4.0 (2018)	7138	7.67
	Amazon FreeRTOS v. 201908	15,413	6.22
	Amazon FreeRTOS v. 202007	12,535	6.1
Flawfinder	Amazon FreeRTOS v. 1.0.0 (2017)	1746	4.34
	Amazon FreeRTOS v. 1.4.0 (2018)	4312	4.63
	Amazon FreeRTOS v. 201908	16,792	6.78
	Amazon FreeRTOS v. 202007	11,724	5.71
RATS	Amazon FreeRTOS v. 1.0.0 (2017)	315	0.78
	Amazon FreeRTOS v. 1.4.0 (2018)	682	0.73
	Amazon FreeRTOS v. 201908	861	0.35
	Amazon FreeRTOS v. 202007	421	0.21

**Table 8:** Total CWEs of IoT OSs by Cppcheck 2.1.

CWE-ID	RIOT	Contiki	FreeRTOS	Amazon FreeRTOS	Totals	Frequency%
CWE-561	3555	2078	25,879	20,322	51,834	53.769%
CWE-398	472	2781	9050	9192	21,495	22.297%
CWE-563	339	1243	6369	5466	13,417	13.918%
CWE-686	67	770	818	548	2203	2.285%
CWE-570	133	272	625	606	1636	1.697%
CWE-476	68	111	790	612	1581	1.640%
CWE-571	68	122	523	363	1076	1.116%
CWE-758	35	132	430	175	772	0.801%
CWE-457	4	46	173	396	619	0.642%
CWE-664	3	1	300	13	317	0.329%
CWE-783	1	60	118	117	296	0.307%
CWE-665	8	40	43	108	199	0.206%
CWE-190	3	3	123	7	136	0.141%
CWE-467	2	50	15	65	132	0.137%
CWE-788	8	25	54	25	112	0.116%
CWE-682	6	0	73	33	112	0.116%
CWE-477	8	21	6	65	100	0.104%
CWE-685	0	68	3	0	71	0.074%
CWE-775	0	16	33	5	54	0.056%
CWE-369	21	0	4	8	33	0.034%
CWE-683	0	4	22	1	27	0.028%
CWE-401	8	0	3	15	26	0.027%
CWE-704	0	0	24	0	24	0.025%
CWE-562	4	0	0	17	21	0.022%
CWE-119	0	20	0	0	20	0.021%
CWE-252	0	4	11	4	19	0.020%
CWE-475	6	9	0	4	19	0.020%
CWE-628	0	0	0	12	12	0.012%
CWE-672	0	0	0	12	12	0.012%
CWE-687	0	0	8	0	8	0.008%
CWE-786	6	0	0	0	6	0.006%
CWE-415	0	0	0	4	4	0.004%
CWE-768	0	4	0	0	4	0.004%
CWE-762	0	0	0	4	4	0.004%

**Table 9:** Total CWEs of IoT OSs by Flawfinder 2.0.11.

CWE-ID	RIOT	Contiki	FreeRTOS	Amazon FreeRTOS	Totals	Frequency %
(CWE-119!/CWE-120)	3129	4703	35,276	18,820	61,928	61.617%
CWE-120	2126	3540	5486	8993	20,145	20.044%
CWE-126	736	1175	2597	2891	7399	7.362%
CWE-134	172	1139	1682	955	3948	3.928%
(CWE-120, CWE-20)	415	397	252	769	1833	1.824%
CWE-190	836	143	72	406	1457	1.450%
CWE-362	106	257	454	566	1383	1.376%
CWE-78	55	69	303	437	864	0.860%
CWE-327	194	22	148	421	785	0.781%
CWE-676	57	113	133	121	424	0.422%
(CWE-362/CWE-367!)	8	13	73	80	174	0.173%
(CWE-807, CWE-20)	4	3	0	61	68	0.068%
CWE-377	3	3	0	42	48	0.048%
(CWE-829, CWE-20)	0	19	0	0	19	0.019%
(CWE-362, CWE-20)	6	0	0	2	8	0.008%
CWE-807	6	0	0	2	8	0.008%
CWE-732	6	0	0	2	8	0.008%
(CWE-120/CWE-785!)	0	0	0	2	2	0.002%
(CWE-250, CWE-22)	0	0	0	2	2	0.002%
(CWE-676, CWE-120, CWE-20)	0	0	0	2	2	0.002%

**Table 10:** Total CWEs of IoT OSs by RATS 2.4.

CWE-ID	RIOT	Contiki	FreeRTOS	Amazon FreeRTOS	Totals	Frequency %
CWE-119	1110	1477	617	1434	4638	55.819%
CWE-120	159	742	407	462	1770	21.302%
CWE-134	578	159	374	139	1250	15.044%
CWE-831	12	119	0	7	138	1.661%
CWE-327	1	21	46	51	119	1.432%
CWE-78	3	64	16	10	93	1.119%
CWE-20	4	51	0	17	72	0.867%
CWE-476	0	0	0	52	52	0.626%
CWE-807	4	3	0	41	48	0.578%
CWE-244	2	1	6	29	38	0.457%
CWE-350	0	0	20	16	36	0.433%
CWE-367	6	6	0	12	24	0.289%
CWE-829	0	19	0	0	19	0.229%
CWE-377	0	3	0	3	6	0.072%
CWE-226	0	0	0	6	6	0.072%

**Table 11:** CodeScene analysis results for RIOT.

IoT OS	Hotspots %	Development Effort in Red Hotspots %	Estimated Bug Fixes in Red Hotspots %	Code Health	Qualitative Team Experience (Months)
RIOT R. 2015.09	1.2%	8%	14%	9.31%	120
RIOT R. 2017.07	1.1%	3%	9%	8.08%	200
RIOT R. 2019.07	0.9%	5%	4%	6.44%	370
RIOT R. 2020.04	0.7%	4%	3%	6.38%	385

**Table 12:** CodeScene analysis results for Contiki.

IoT OS	Hotspots %	Development Effort in Red Hotspots %	Estimated Bug Fixes in Red Hotspots %	Code Health	Qualitative Team Experience (Months)
Contiki R. 2.4	2.5%	6%	17%	4.77%	170
Contiki R. 2.7	2.3%	3%	11%	4.33%	128
Contiki R. 3.0	2.1%	4%	20%	4.24%	200
Contiki R. 3.1	2%	5%	16%	4.07%	295

**Table 13:** Correlation coefficient of evolutionary properties and the presence of IoT OS vulnerabilities.

OS	X	Y	Correlation Coefficient (Cppcheck)	Correlation Coefficient (Flawfinder)	Correlation Coefficient (RATS)
RIOT	Total of security errors	SLOC	0.994	0.993	0.302
	Total of security errors	Code Health	−0.971	−0.970	−0.445
	Errors per 1K SLOC	Code Hotspots	0.774	0.825	0.845
	Errors per 1K SLOC	Qualitative Team Experience	−0.808	−0.862	−0.850
Contiki	Total of security errors	SLOC	0.961	0.961	0.952
	Total of security errors	Code Health	−0.998	−0.997	−0.998
	Errors per 1K SLOC	Code Hotspots	0.816	0.977	0.833
	Errors per 1K SLOC	Qualitative Team Experience	−0.991	−0.876	−0.946

## Data Availability

The data presented in this study is contained within the article.

## Appendix A

CWE-ID	Description
CWE-561	Dead Code
CWE-398	Seven Pernicious Kingdoms (7PK) vulnerability—Code Quality
CWE-563	Assignment to Variable without Use
CWE-686	Function Call with Incorrect Argument Type
CWE-570	Expression is Always False
CWE-476	NULL Pointer Dereference
CWE-571	Expression is Always True
CWE-758	Reliance on Undefined, Unspecified, or Implementation-Defined Behavior
CWE-457	Use of Uninitialized Variable
CWE-664	Improper Control of a Resource Through its Lifetime
CWE-783	Operator Precedence Logic Error
CWE-665	Improper Initialization
CWE-190	Integer Overflow or Wraparound
CWE-467	Use of *sizeof()* on a Pointer Type
CWE-ID	Description
CWE-788	Access of Memory Location After End of Buffer
CWE-682	Incorrect Calculation
CWE-477	Use of Obsolete Function
CWE-685	Function Call with Incorrect Number of Arguments
CWE-775	Missing Release of File Descriptor or Handle after Effective Lifetime
CWE-401	Missing Release of Memory after Effective Lifetime
CWE-683	Function Call with Incorrect Order of Arguments
CWE-369	Divide By Zero
CWE-704	Incorrect Type Conversion or Cast
CWE-562	Return of Stack Variable Address
CWE-475	Undefined Behavior for Input to API
CWE-119	Improper Restriction of Operations within the Bounds of a Memory Buffer
CWE-252	Unchecked Return Value
CWE-628	Function Call with Incorrectly Specified Arguments
CWE-672	Operation on a Resource after Expiration or Release
CWE-687	Function Call with Incorrectly Specified Argument Value
CWE-786	Access of Memory Location Before Start of Buffer
CWE-415	Double Free
CWE-768	Incorrect Short Circuit Evaluation
CWE-762	Mismatched Memory Management Routines
CWE-120	Buffer Copy without Checking Size of Input (Classic Buffer Overflow)
CWE-134	Use of Externally Controlled Format String
CWE-831	Signal Handler Function Associated with Multiple Signals
CWE-327	Use of a Broken or Risky Cryptographic Algorithm
CWE-78	Improper Neutralization of Special Elements used in an OS Command
CWE-20	Improper Input Validation
CWE-807	Reliance on Untrusted Inputs in a Security Decision
CWE-244	Improper Clearing of Heap Memory Before Release (‘Heap Inspection’)
CWE-350	Reliance on Reverse DNS Resolution for a Security-Critical Action
CWE-367	Time-of-check Time-of-use (TOCTOU) Race Condition
CWE-829	Inclusion of Functionality from Untrusted Control Sphere
CWE-377	Insecure Temporary File
CWE-226	Sensitive Information in Resource Not Removed Before Reuse
CWE-126	Buffer Over-read
CWE-362	Concurrent Execution using Shared Resource with Improper Synchronization
CWE-676	Use of Potentially Dangerous Function
CWE-732	Incorrect Permission Assignment for Critical Resource
CWE-785	Use of Path Manipulation Function without Maximum-sized Buffer
CWE-250	Execution with Unnecessary Privileges
CWE-22	Improper Limitation of a Pathname to a Restricted Directory
(CWE-119!/CWE-120)	The vulnerability is mapped to CWE-119 and that CWE-120 is a subset of CWE-119
(CWE-120, CWE-20)	Classic Buffer Overflow and Improper Input Validation
(CWE-362/CWE-367!)	The vulnerability is mapped to CWE-367, a subset of CWE-362
(CWE-362, CWE-20)	The vulnerability is mapped to CWE-362 and CWE-20
(CWE-807, CWE-20)	The vulnerability is mapped to CWE-807 and CWE-20
(CWE-829, CWE-20)	The vulnerability is mapped to CWE-829 and CWE-20
(CWE-120/CWE-785!)	The vulnerability is mapped to CWE-785, a subset of CWE-120
(CWE-250, CWE-22)	The vulnerability is mapped to CWE-250 and CWE-22
(CWE-676, CWE-120, CWE-20)	The vulnerability is mapped to CWE-676, CWE-120 and CWE-20
